# Further evaluation and validation of the VETSCAN IMAGYST: in-clinic feline and canine fecal parasite detection system integrated with a deep learning algorithm

**DOI:** 10.1186/s13071-021-04591-y

**Published:** 2021-01-29

**Authors:** Yoko Nagamori, Ruth Hall Sedlak, Andrew DeRosa, Aleah Pullins, Travis Cree, Michael Loenser, Benjamin S. Larson, Richard Boyd Smith, Cory Penn, Richard Goldstein

**Affiliations:** 1 Department of Veterinary Pathobiology, College of Veterinary Medicine, Oklahoma State University, Stillwater, OK 74078 USA; 2 Veterinary Medicine Research and Development, Zoetis, 333 Portage Street, Kalamazoo, MI 49007 USA; 3 Global Diagnostics, Zoetis, 10 Sylvan Way, Parsippany, NJ 07054 USA; 4 Petcare, Zoetis, 10 Sylvan Way, Parsippany, NJ 07054 USA; 5 Techcyte, Incorporated, 384 S 400 W #125, Lindon, UT 84042 USA

**Keywords:** Deep learning, Fecal egg identification, Veterinary parasitology diagnostic, *Ancylostoma*, *Toxocara cati*, *Cystoisospora*, *Giardia*, Oocyst, Cyst

## Abstract

**Background:**

Fecal examinations in pet cats and dogs are key components of routine veterinary practice; however, their accuracy is influenced by diagnostic methodologies and the experience level of personnel performing the tests. The VETSCAN IMAGYST system was developed to provide simpler and easier fecal examinations which are less influenced by examiners’ skills. This system consists of three components: a sample preparation device, an automated microscope scanner, and analysis software. The objectives of this study were to qualitatively evaluate the performance of the VETSCAN IMAGYST system on feline parasites (*Ancylostoma* and *Toxocara cati*) and protozoan parasites (*Cystoisospora* and *Giardia*) and to assess and compare the performance of the VETSCAN IMAGYST centrifugal flotation method to reference centrifugal and passive flotation methods.

**Methods:**

To evaluate the diagnostic performance of the scanning and algorithmic components of the VETSCAN IMAGYST system, fecal slides were prepared by the VETSCAN IMAGYST centrifugal flotation technique with pre-screened fecal samples collected from dogs and cats and examined by both an algorithm and parasitologists. To assess the performance of the VETSCAN IMAGYST centrifugal flotation technique, diagnostic sensitivity and specificity were calculated and compared to those of conventional flotation techniques.

**Results:**

The performance of the VETSCAN IMAGYST algorithm closely correlated with evaluations by parasitologists, with sensitivity of 75.8–100% and specificity of 93.1-100% across the targeted parasites. For samples with 50 eggs or less per slide, Lin’s concordance correlation coefficients ranged from 0.70 to 0.95 across the targeted parasites. The results of the VETSCAN IMAGYST centrifugal flotation method correlated well with those of the conventional centrifugal flotation method across the targeted parasites: sensitivity of 65.7–100% and specificity of 97.6–100%. Similar results were observed for the conventional passive flotation method compared to the conventional centrifugal flotation method: sensitivity of 56.4–91.7% and specificity of 99.4–100%.

**Conclusions:**

The VETSCAN IMAGYST scanning and algorithmic systems with the VETSCAN IMAGYST fecal preparation technique demonstrated a similar qualitative performance to the parasitologists’ examinations with conventional fecal flotation techniques. Given the deep learning nature of the VETSCAN IMAGYST system, its performance is expected to improve over time, enabling it to be utilized in veterinary clinics to perform fecal examinations accurately and efficiently.
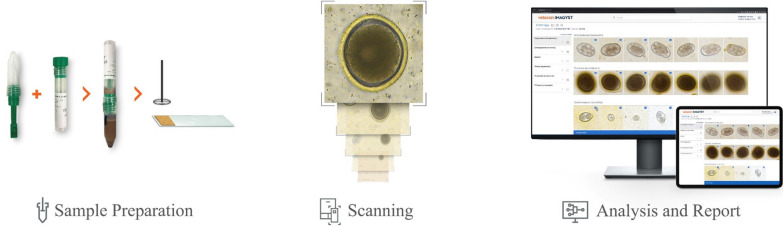

## Background

Domestic cat ownership in the USA has been increasing at the same time as an overall rise in pet ownership. In 2017–2018, up to 47.1 million households in the USA owned at least one cat, with 76% of owners considering their cats to be “family members” [1]. However, cats are generally less medicalized or served by veterinarians compared to dogs [2], even though studies have reported that gastrointestinal parasitism in cats is common [3–8]. A recent retrospective study demonstrated a significant increase in the prevalence of intestinal parasitic infections in client-owned cats during a 12-year period (19.0% in 2007 vs. 32.5% in 2018; *P* < 0.0001) [7]. The majority of people acquire cats from shelters or as rescues and through adoption of strays [1], although these cat populations harbor a high prevalence of parasitic infections, ranging between 31.8 and 67.2% [3–5, 7, 8]. The most prevalent nematodes identified in cats are *Ancylostoma* and *Toxocara cati* [3–9]. The main species of feline *Ancylostoma* found in North America are *Ancylostoma tubaeforme* and *Ancylostoma braziliense* [10]*.* Since feline *Ancylostoma* and *T. cati* are zoonotic parasites [11–18], it is important to conduct routine fecal examinations of cats, and treat them as necessary to maintain their wellness and that of their owners.

The most commonly detected protozoan parasites in domestic cats and dogs in North America are *Cystoisospora* (formally *Isospora*) and *Giardia* [3, 5–8, 19–25]. Studies evaluating feline fecal samples have reported the prevalence of *Cystoisospora* and *Giardia* to range between 3.8–26.0% and 1.2–9.9%, respectively, with the highest prevalence in shelter cats [3, 5–8, 19, 20, 24, 25]. The prevalence of *Cystoisospora* and *Giardia* in dogs has been reported to range between 0.5–10.4% and 3.3–13%, respectively [8, 19, 21–24, 26–31]. *Cystoisospora* oocysts and *Giardia* cysts/trophozoites are small in size and can be challenging to detect by microscopy, especially when fecal samples contain a low number of oocysts, cysts, or trophozoites. In addition, intermittent shedding of *Giardia* cysts/trophozoites makes it more difficult to diagnose giardiasis [10, 32–35].

Fecal examinations in dogs and cats to detect evidence of gastrointestinal parasitism are widely recognized as an important component of routine veterinary care, both for maintaining pet health and for identifying parasites of zoonotic significance [36]. However, the accuracy and usefulness of fecal testing can be influenced by many factors, such as differences in methodology and the experience level of personnel conducting the tests [36–38]. Of note, *Giardia* has been identified as a particularly difficult parasite to diagnose by coprology in veterinary practice [36]. Computer-based algorithms to identify parasites in fecal examinations have recently been developed and introduced with the aim of improving the accuracy and consistency of diagnosing parasitic diseases in dogs and cats [39–42].

**Fig. 1:**
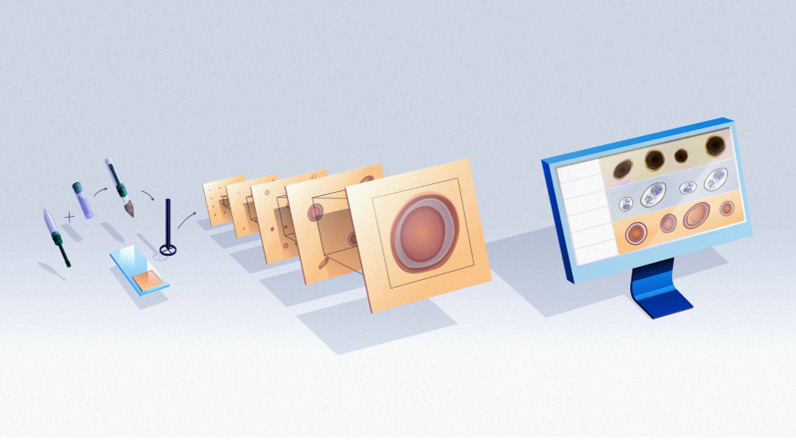
The VETSCAN IMAGYST system consists of three main components: a sample preparation device, a digital microscope scanner, and data analysis and reporting

**Fig. 2:**
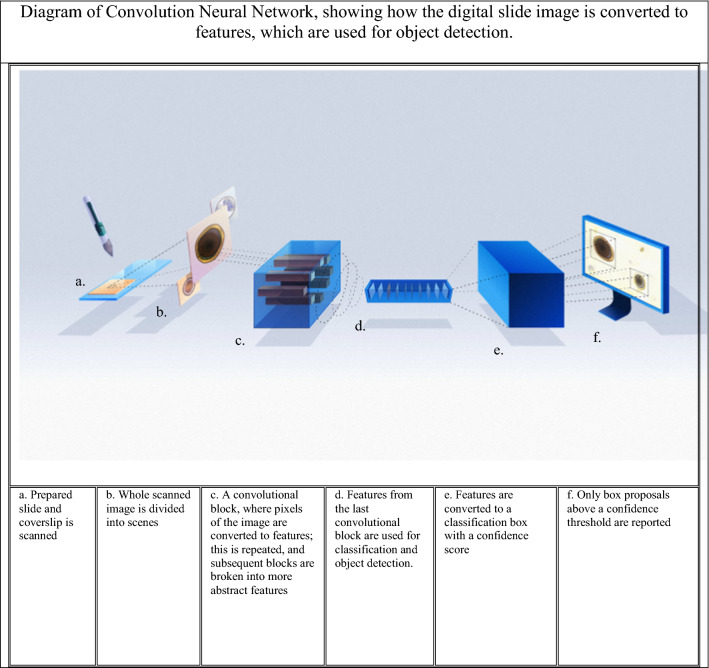
Diagram of the VETSCAN IMAGYST algorithm’s convolution neural network and object detection process

The novel VETSCAN IMAGYST system evaluates fecal samples for evidence of parasitic infections in an organized and uncomplicated fashion that does not depend greatly on an examiner’s level of experience. This system was able to reliably detect four targeted parasites (genera *Ancylostoma*, *Toxocara*, *Trichuris*, and family Taeniidae) in fecal samples of 84 dogs and 16 cats, and the results closely correlated with those reported by a parasitologist following fecal examination of the animals (Pearson correlation coefficients 0.83–0.99 across the four targeted parasites) [40]. The VETSCAN IMAGYST system consists of three main components: a sample preparation device, an automated commercially available microscope scanner, and a data analysis process using deep neural networks (Fig. 1). The VETSCAN IMAGYST system applies a deep learning object detection algorithm which uses a convolutional neural network to identify convolutional layers that automatically learn the most discriminating features between classes (Fig. 2). The algorithm assigns a probability score to each image recognized within each sample preparation as being that of an egg belonging to a parasite genus/group that the software has been previously trained/calibrated to recognize. A mature algorithm model, which can perceive and distinguish the morphology of individual parasite eggs and non-parasite objects on fecal flotation slides, is developed through a training process that utilizes samples characterized by an expert. Once the model is sufficiently mature, it is tested against several evaluation datasets to ensure that the final most promising model will be generalizable to other similar domains.

The present study is an extension of a previously published VETSCAN IMAGYST study [40], with two additional main objectives. First, to qualitatively evaluate the diagnostic performance of the VETSCAN IMAGYST system to recover and correctly identify feline *Ancylostoma* eggs, *Toxocara cati* eggs, *Cystoisospora* oocysts, and *Giardia* cysts in feces of naturally infected cats and dogs, compared to manual identification by experts with conventional sample preparation methods. Second, to compare the diagnostic performance of the VETSCAN IMAGYST centrifugal preparation method to those of standard centrifugal and passive flotation methods for the four targeted parasites: feline *Ancylostoma*, *Toxocara cati*, *Cystoisospora*, and *Giardia*.

## Methods

### Fecal sample collections

Fecal samples from client-owned and shelter cats and dogs submitted to the Oklahoma Animal Disease Diagnostic Laboratory of Oklahoma State University were processed for egg, oocyst, and cyst recovery using a Wisconsin fecal egg counting method, as previously described [10, 40]. A total of 100 fecal samples were collected weighing a minimum of 8 g and confirmed positive for at least one of the targeted parasites: (1) feline *Ancylostoma*, (2) *Toxocara cati*, (3) feline *Cystoisospora*, and (4) canine and feline *Giardia*; as well as 10 feline samples negative for any targeted parasites. Samples were included as positives or negatives for the relevant analyses; samples containing multiple targeted parasites were counted as a positive for more than one analysis, and some samples were counted as a negative for more than one analysis. For the *Cystoisospora* analysis, 100 additional fecal samples from a second collection separated from the first by several months, with the same sample acquisition criteria including canine samples, were included. Table 1 summarizes the number of screened positive and negative samples assessed for each evaluation. Different reference methods were used for the VETSCAN IMAGYST algorithm and sample preparation assessments; therefore, the total number of positives and negatives varies between evaluations. To maintain morphological integrity of the diagnostic forms of parasite elements, all samples were preserved at 4 °C until the study. For 38 samples, a low level of formalin solution was added as a fixative to ensure integrity of the parasite elements.

**Table 1:** Summary of fecal samples included in this study by targeted parasites based upon initial characterization

Targeted parasite	Samples (*n*)	Flotation solution utilized for sample preparation	Sample sizes			
			Positive	Negative	Total	Mixed infections^a^
*Ancylostoma*	Feline (80)	Sugar	20	60	80	21
*Toxocara cati*	Feline (80)	Sugar	34	46	80	21
*Cystoisospora*	Feline (96)	Sugar	36	164	200	72
	Canine (104)					
*Giardia*	Feline (80)	Zinc sulfate/sugar^b^	39	61	100	28
	Canine (20)					

^a^Mixed infections are samples that were positive for more than 1 targeted parasite

^b^Zinc sulfate solution was used for* Giardia* samples; sugar solution was used for the rest of the samples

### VETSCAN IMAGYST scanner and algorithm

The VETSCAN IMAGYST system has previously been described [39]. Briefly, slides with fecal samples were read by the Motic EasyScan One digital slide scanner (Motic, Kowloon Bay, Hong Kong), which provided 40× effective resolution. The scanned images were then automatically uploaded and analyzed in the cloud with an updated deep learning objective detection algorithm, version 3033, which was developed based on the You only look once (YOLOv3) model [43] using the adaptive moment estimation (Adam; an algorithm for first-order gradient-based optimization of stochastic objective functions) optimizer [44] (Techcyte, Lindon, UT). Version 3033 was selected after several trainings of eight epochs with approximately 2,000–5,500 images that were previously captured by the VETSCAN IMAGYST system and reserved to improve the algorithm performance. Twice during each epoch, a 416 × 416-pixel area was randomly translated around every label to be used for further trainings. After localization and classification of the objects of interest, the resulting images were then available for viewing on the VETSCAN IMAGYST platform, and a downloadable portable document format report was generated (Fig. 1). The VETSCAN IMAGYST analysis software also has the quantitative ability to count the parasites of interest. In the present study, this quantitative capability was partly evaluated in the samples with ≤ 50 parasite elements/g of feces, as our main objective was to qualitatively evaluate the diagnostic performance of the VETSCAN IMAGYST system recovering and correctly identifying targeted parasites.

### Assessment of VETSCAN IMAGYST algorithm performance

**Fig. 3:**
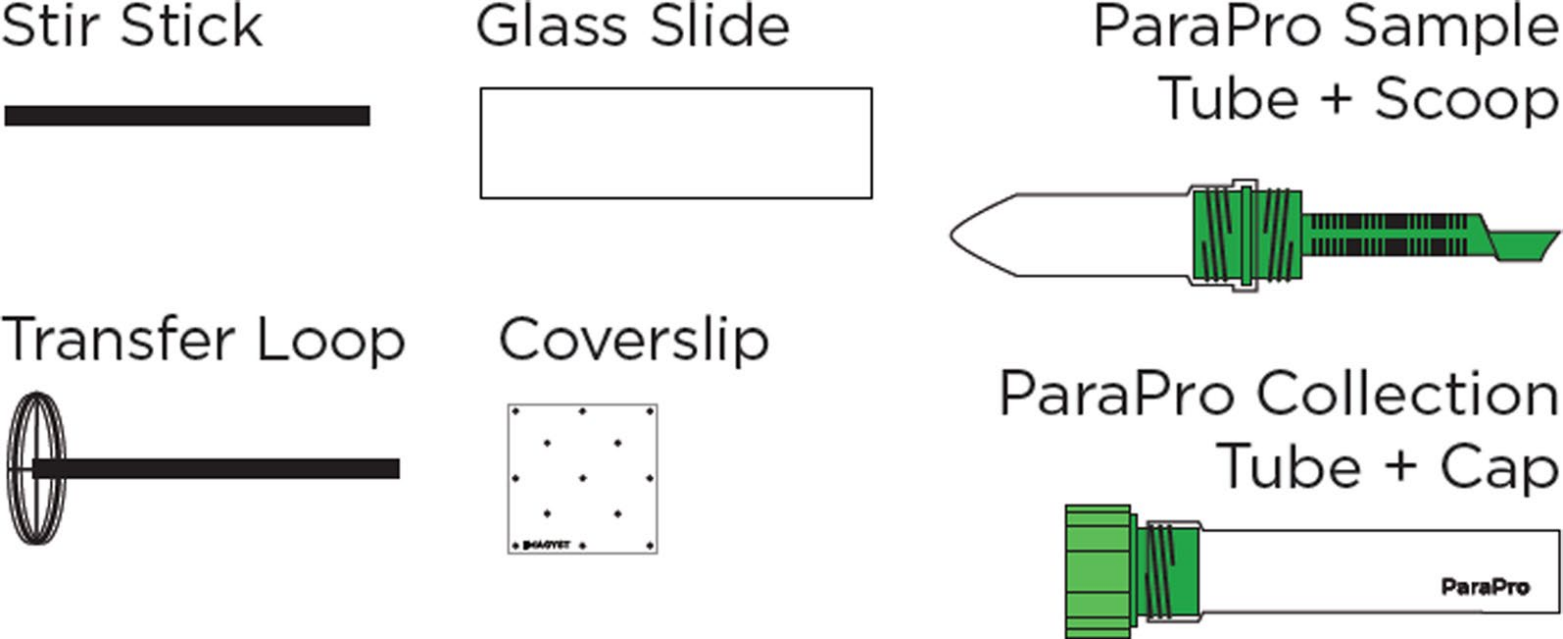
VETSCAN IMAGYST sample preparation materials

To qualitatively evaluate the ability of the scanning and algorithmic components of the VETSCAN IMAGYST system to identify diagnostic forms of the targeted parasites, slides were prepared with the VETSCAN IMAGYST centrifugal flotation technique for each pre-screened fecal sample, as described previously by Nagamori et al. [40]. Briefly, pre-screened fecal samples were examined randomly. The VETSCAN IMAGYST fecal preparation device, which was specifically redesigned and produced from Apacor mini Parasep SF (Apacor, Wokingham, UK), was utilized to perform the VETSCAN IMAGYST centrifugal method. The VETSCAN IMAGYST fecal preparation device consists of two tubes: the sample tube with a sample scoop, which can hold approximately 1 g of feces; and the collection tube containing two types of flotation solution, zinc sulfate solution (specific gravity, 1.18) for *Giardia* samples and sugar solution (specific gravity, 1.25) for the rest of the samples, including *Ancylostoma*, *Toxocara*, *Cystoisospora*, and negatives. Sugar solution was selected for the current analysis as it is more effective than some of the salt solutions used to float dense parasite eggs, is inexpensive, and allows easier maintenance of fecal slides since it does not crystalize quickly [10]. The sample tube and collection tube were firmly screwed together, shaken and centrifuged for 2 min at 300-500 relative centrifugal force (rcf). Following centrifugation, the sample tube was unscrewed from the collection tube. The transfer loop was used to collect diagnostic forms of parasites from the top of the flotation solution and transfer them to a microscope slide (Fig. 3). The coverslip marked with the IMAGYST label was placed such that the IMAGYST label could be read correctly, ensuring that the marked coverslip was accurately placed on the slide. The slide was placed in a slide tray, which was then inserted into an automated microscope scanner, and the resulting digitally scanned image uploaded to a cloud-based server for analysis and result generation.

The VETSCAN IMAGYST fecal preparation was performed by two technicians. After the slides were analyzed by the VETSCAN IMAGYST system, two parasitologists and one technician, all well experienced in diagnostic parasitology, evaluated each slide microscopically at 100×, 200×, and 400× magnification. Identification of the parasites was based on the morphology of eggs, oocysts, and cysts [10]. During the manual examinations by the parasitologists and technician, counts of the entire slide were performed when up to 50 eggs, oocysts, or cysts per each targeted parasite were present. The numbers were estimated where burdens were high (> 50) and, for eggs, were recorded as “medium” for slides with 51-250 eggs per gram (EPG), and “high” for slides with > 250 EPG. The VETSCAN IMAGYST algorithm, on the other hand, provided counts regardless of egg burden. Results from the VETSCAN IMAGYST algorithm and microscopic examinations by experts were compared and statistically analyzed. For *Giardia* fecal samples, an additional analysis of the subset of samples with > 10 cysts per gram (CPG) was conducted.

### Assessment of sample preparation methods

Performance (sensitivity and specificity) of the VETSCAN IMAGYST centrifugal technique was assessed by comparing it to conventional centrifugal flotation and passive flotation methods using visual microscopy. For each fecal sample, slides were prepared by 3 different sample preparation techniques: VETSCAN IMAGYST centrifugal flotation, conventional centrifugal flotation, and conventional passive flotation using the OVASSAY Plus Kit Fecal Flotation Device (Zoetis, Parsippany Troy Hills, NJ). Two technicians prepared the slides for the VETSCAN IMAGYST centrifugal flotation technique as described above. The process for the reference centrifugal flotation technique has previously been described [40]. Briefly, 33% zinc sulfate solution (specific gravity, 1.18) for *Giardia* samples and Sheather’s sugar solution (specific gravity, 1.25) for the rest of the samples were used to suspend 1 g of feces, which was then strained and placed in a 15-ml centrifuge tube. Flotation solution was added to the tube until a convex meniscus was formed, then a coverslip was added, and the samples centrifuged in a Centra CL2 centrifuge (Thermo Fisher Scientific, Waltham, MA) at approximately 440 rcf for 5 min. After removing the coverslip and placing it on a glass slide, a microscopic examination was performed [10]. The OVASSAY Plus Kit Fecal Flotation Device with 33% zinc sulfate solution (specific gravity, 1.18) was used to perform the passive fecal flotation test, following the manufacturer’s instructions [45]. Several student workers prepared the fecal slides with conventional centrifugal and passive fecal flotation methods, and all the slides were microscopically examined by three experts as described above. The duration of the microscopic examinations by the experts was not restricted or recorded. The diagnostic performance of the VETSCAN IMAGYST centrifugal flotation method was compared with both reference methods, conventional centrifugal and OVASSAY passive flotation.

### Statistical analysis

Samples for which any parasite elements were observed, or for which in a separate analysis > 10 CPG for *Giardia* were recorded, were considered positive. Two by two tables were constructed, and sensitivity and specificity calculated with 95% Jeffreys confidence interval estimates. Lin’s concordance correlation coefficients (ρc) were calculated when ≤ 50 eggs, oocysts or cysts per slide were counted microscopically by the three experts. When > 50 eggs/oocysts/cysts were estimated per slide, the data were not included in the correlation analysis. SAS version 9.4M6 (SAS Institute, Cary, NC) was used for the statistical analysis.

## Results

### Algorithm performance

**Fig. 4:**
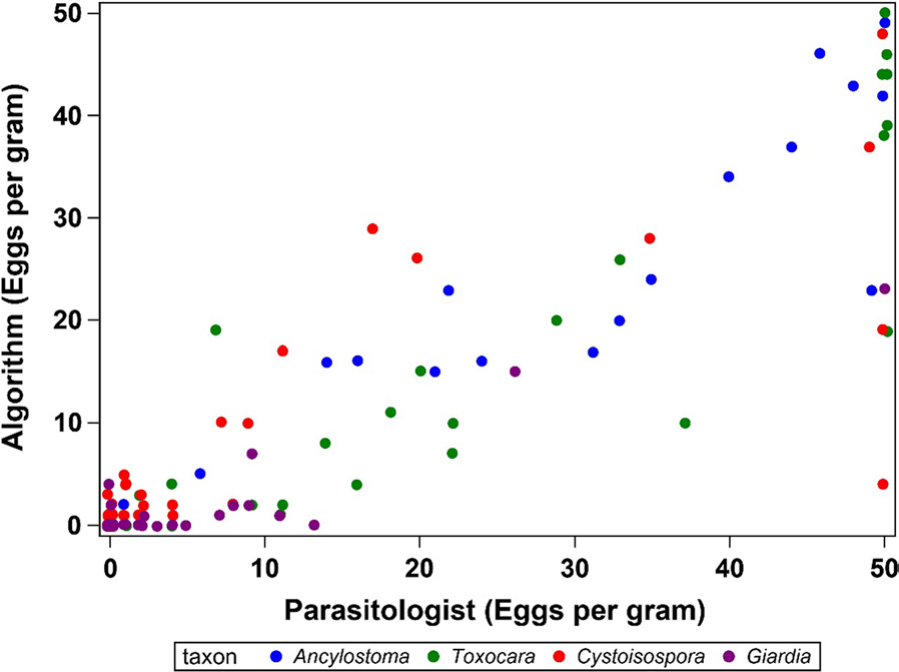
Algorithm performance analysis: quantitative visualization of egg identification [eggs per gram (EPG)] by an expert parasitologist (*x*-axis) and the VETSCAN IMAGYST scanner/algorithm (*y*-axis) for the samples with ≤ 50 EPG prepared by the VETSCAN IMAGYST centrifugal flotation method

Comparisons between the results generated by the VETSCAN IMAGYST system and those recorded by the experts were made to assess the performance of the VETSCAN IMAGYST scanner and algorithm in the identification of eggs, oocysts, and cysts of targeted parasites (Table 2 and Fig. 4). The diagnostic sensitivity and specificity of the VETSCAN IMAGYST system in comparison to the expert assessments ranged from 75.8–100% and 93.1–100%, respectively, for the four targeted parasites. Twelve of 33 (36%) *Giardia* samples contained a very low number of cysts, i.e. ≤ 10 CPG. The diagnostic sensitivity for *Giardia* dramatically increased to 95.2% (95% confidence interval: 79.8–99.5%) after the *Giardia* samples with ≤ 10 CPG were excluded from the analysis. Quantitative comparisons for samples with ≤ 50 EPG, OPG, or CPG were performed, and the VETSCAN IMAGYST diagnostic results closely matched those of the experts for each targeted parasite (ρc of 0.95 for feline *Ancylostoma*, 0.89 for *Toxocara cati*, 0.80 for *Cystoisospora*, and 0.70 for *Giardia*).

**Table 2:** Algorithm performance analysis: diagnostic sensitivity and specificity comparing the results reported by an expert (reference) versus by the VETSCAN IMAGYST scanner and algorithm for samples prepared with the VETSCAN IMAGYST centrifugal flotation method

	Feline *Ancylostoma*	*Toxocara cati*	*Cystoisospora*	*Giardia*
True positive	19	32	26	25
False positive	1	0	12	2
True negative	60	46	161	65
False negative	0	2	1	8
Total	80	80	200	100
Sensitivity (95% confidence interval)	100 (87.8–100)	94.1 (82.4–98.8)	96.3 (84.0–99.6)	75.8 (59.4–87.8)^a^
Specificity (95% confidence interval)	98.4 (92.6–99.8)	100 (94.7–100)	93.1 (88.6–96.2)	97.0 (90.8–99.4)

^a^Thirty-six percent (12/33) of *Giardia* samples contained ≤ 10 cysts per gram (CPG); diagnostic sensitivity of *Giardia* samples with > 10 CPG increased to 95.2% (95% confidence interval: 79.8-99.5%)

### Sample preparation performance

When the performance of the VETSCAN IMAGYST centrifugal flotation (test method) and the conventional centrifugal flotation (reference method) were compared, the diagnostic sensitivity and specificity of the VETSCAN IMAGYST ranged from 65.7–100% and 97.6–100%, respectively, across the four targeted parasites (Table 3). When the sample preparation performance of the OVASSAY passive flotation method was compared to the conventional centrifugation flotation method, the diagnostic sensitivity and specificity of the former ranged from 56.4–91.7% and 99.4–100%, respectively (Table 3).

**Table 3:** Sample preparation analysis: diagnostic sensitivity and specificity comparing the VETSCAN IMAGYST centrifugal flotation (*VS-Ic*) and OVASSAY passive flotation (*O-pf*) methods versus a conventional centrifugal flotation method (reference); all slides were read by an expert

	Feline *Ancylostoma*		*Toxocara cati*		*Cystoisospora*^a^		*Giardia*	
	*n* = 80		*n* = 80		*n* = 200		*n* = 100	
	VS-Ic	O-pf	VS-Ic	O-pf	VS-Ic	O-pf	VS-Ic	O-pf
True positive	19	17	34	33	23	22	32	22
False positive	0	0	0	0	4	1	1	0
True negative	61	61	44	44	161	164	60	61
False negative	0	2	2	3	12	13	7	17
Sensitivity (95% confidence interval)	100 (87.8–100)	89.5 (70.3–97.7)	94.4 (83.4–98.8)	91.7 (79.4–97.6)	65.7 (49.2–79.7)	62.9 (46.3–77.3)	82.1 (68.0–91.6)	56.4 (40.9–71.1)
Specificity (95% confidence interval)	100 (96.0–100)	100 (96.0–100)	100 (94.5–100)	100 (94.5–100)	97.6 (94.3–99.2)	99.4 (97.2–99.9)	98.4 (92.6–99.8)	100 (96.0–100)

^a^*Cystoisospora* results are a combination of 2 independent studies with the same sample acquisition criteria. *Cystoisospora* species included *Cystoisospora canis, Cystoisospora ohioiensis, Cystoisospora felis* and *Cystoisospora rivolta*

## Discussion

This is the second study demonstrating that the VETSCAN IMAGYST system integrated with a deep learning object detection algorithm can successfully recognize and identify diagnostic forms of gastrointestinal parasites in dogs and cats on fecal flotation slides scanned by an automated microscope. Whereas our previous study evaluated the VETSCAN IMAGYST system for the detection of eggs of *Ancylostoma*, *Toxocara*, *Trichuris*, and Taeniidae in 84 canine and 16 feline fecal samples [40], this study assessed the ability of the system to detect eggs of the feline nematodes *Ancylostoma* and *Toxocara cati*, and oocysts of the protozoan parasite *Cystoisospora* and cysts of the protozoan parasite *Giardia*, in 104 canine and 96 feline fecal samples, making it a more comprehensive analysis of this novel system.

**Fig. 5:**
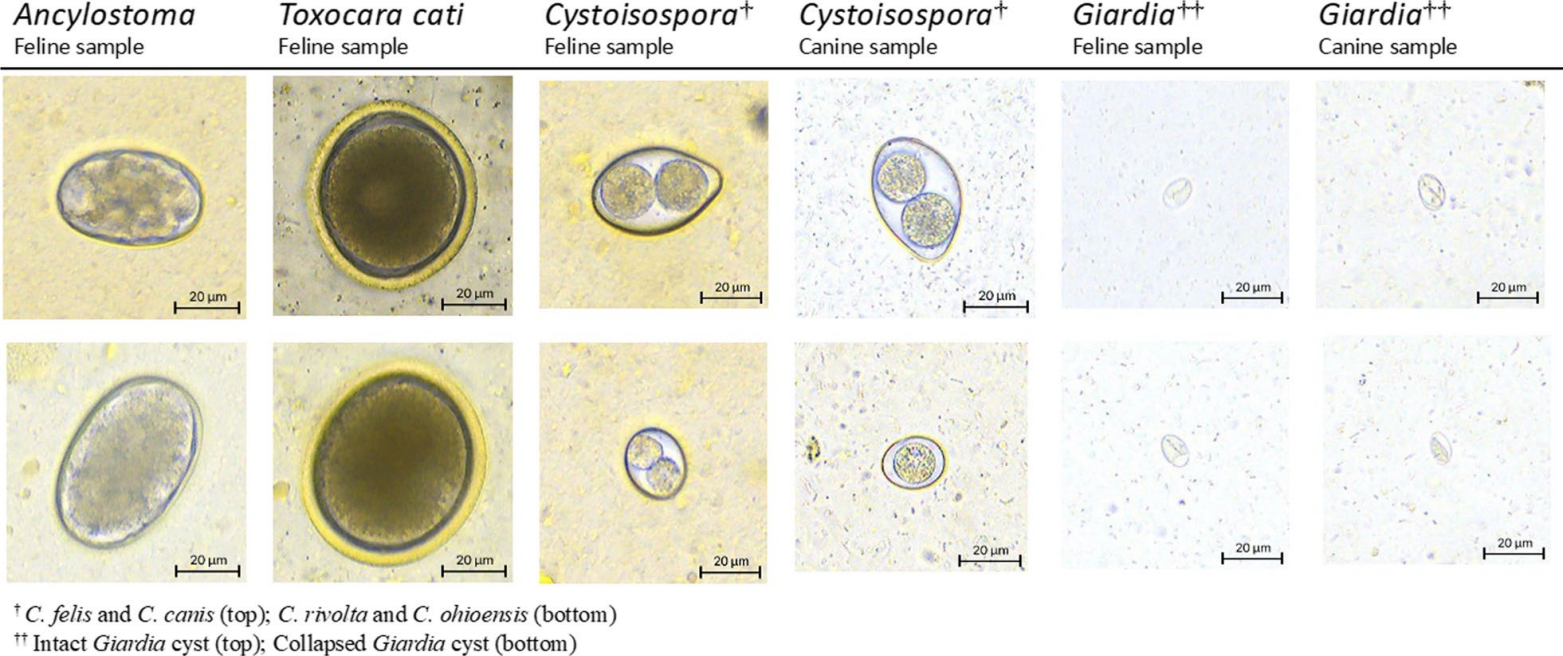
Images of targeted parasites captured by the VETSCAN IMAGYST system

Although both domestic dogs (*Canis lupus familiaris*) and cats (*Felis catus*) belong to the order Carnivora, dogs are classified into the superfamily Canoidea and cats are classified into the superfamily Feloidea [46, 47]. Diets of canids can vary from herbivorous to omnivorous; however, all felids require a strictly carnivorous diet [47]. A high-protein diet of animal origin is essential for domestic cats to obtain some of their nutritional requirements, such as taurine, as well as arachidonic acid and vitamin A [47]. Due to their diet, fecal samples of cats commonly contain a large amount of fat and are soft, sticky and clay-like in consistency, which often makes it more difficult, or sometimes impossible, to read fecal slides since more debris floats with fats, especially when a viscous sugar solution is used in a centrifugal flotation technique. Modification of fecal flotation procedures, involving an initial water wash where the supernatant is discarded after the initial spin and the sediment resuspended with a flotation solution to remove excess fat, mucus, and debris (double centrifugal fecal flotation technique), can be used [10]. In the present study, the VETSCAN IMAGYST centrifugal flotation method recovered parasite elements from feline feces, and the VETSCAN IMAGYST scanner and algorithm successfully captured and identified targeted parasites (Fig. 5).

To our knowledge, this is the first report demonstrating the ability of the VETSCAN IMAGYST system to recover and accurately detect protozoan parasites, i.e. *Cystoisospora* oocysts and *Giardia* cysts. Although coccidiosis is generally considered a self-limiting infection in mature dogs and cats due to their rapid development of immunity [18], *Cystoisospora* is an ubiquitous and important pathogen in puppies and kittens, with infection often resulting in diarrhea, abdominal pain, anorexia, bloody diarrhea, anemia, and even mortality in severe cases [10, 17, 48]. Since *Cystoisospora* undergo fast replication in the pathogenic intestinal stage and a high number of oocysts are excreted in host feces, causing environmental contamination, it is considered critical and thus highly recommended to conduct a fecal examination with centrifugation for puppies and kittens at least four times during the first year of life for treatment of *Cystoisospora* at an early stage of infection [48, 49]. Different species of *Cystoisospora* are commonly diagnosed in dogs and cats: *Cystoisospora canis* and *Cystoisospora ohioensis* in dogs, and *Cystoisospora felis* and *Cystoisospora rivolta* in cats. Oocysts of *C. canis* and *C. felis* are slightly bigger, at approximately 38-51 × 27-39 µm in size, than those of *C. ohioensis* and *C. rivolta*, which are approximately 17-27 x 15-24 µm in size [10]. Due to the smaller size of coccidian oocysts compared to helminth eggs, *Cystoisospora* can be easily overlooked, especially when the number of oocysts on a fecal slide is low and an inaccurate microscopic focus is used for examination. The VETSCAN IMAGYST system correctly identified oocysts of all four *Cystoisospora* species in canine and feline fecal samples and successfully reported them as *Cystoisospora* (coccidia) (Fig. 5).

The diagnostic sensitivity and specificity of the VETSCAN IMAGYST scanner and algorithm for the *Giardia* samples compared with the results reported by the experts were 75.8 and 97.0%, respectively (Table 2). As previously discussed, a common challenge for many object detection algorithm models is to precisely localize and distinguish small objects such as *Giardia* cysts [40]. The nature of a deep learning algorithm, however, means that its performance continues to improve with further training. It is important to note that the diagnostic sensitivity was dramatically increased to 95.2% by removing the 12 *Giardia* samples that had ≤ 10 CPG from the analysis; detecting such a low number of cysts is extremely demanding when slides are examined by visual microscopy. Additionally, the examination and counts of CPG on these *Giardia* slides were carefully performed by a well-trained diagnostic parasitologist with no time limit, which most likely resulted in a much higher diagnostic performance compared to that usually achieved by technicians in daily veterinary practice.

The detection of *Giardia* cysts and triphozoites by microscopic examination is generally considered the most sensitive technique for the diagnosis of giardiasis, and therefore, a great deal of training and experience is required for the confident diagnosis of this disease [10]. It is challenging to identify *Giardia* infection by fecal examination because, in addition to their small size and transparency, *Giardia* cysts and trophozoites are intermittently shed in feces, and multiple fecal examinations may be necessary to rule out infection. Fresh fecal samples, obtained preferably within 30 min of defecation, are often required to detect motile trophozoites; however, *Giardia* cysts and trophozoites are fairly fragile and their shape easily distorted in flotation solution [10]. A 33% zinc sulfate solution (specific gravity, 1.18) is preferred and recommended for the detection of *Giardia* cysts, as other flotation solutions can rapidly cause osmotic damage to them, which increases the difficulty of perceiving them on fecal slides [10, 34, 50, 51]. During the present study, the VETSCAN IMAGYST system effectively recognized and identified both intact and collapsed *Giardia* cysts (Fig. 5). Testing for *Giardia* is recommended not only in symptomatic dogs and cats, but also in dogs and cats newly introduced to homes which have other pets that are free of infection, as many *Giardia* infections can be asymptomatic [10, 32, 48]. Since there is no perfect flotation solution for the recovery of all the different types of parasites [10], it is important to consider the advantages and disadvantages of each individual solution when selecting one for general use. Some experts recommend performing two centrifugal flotation tests by using both Sheather’s sugar and 33% zinc sulfate solutions to achieve a broader range of gastrointestinal parasite detections. In cases where *Giardia* is suspected, analysis using the sugar flotation solution should also be performed on fecal samples to check for other parasites.

The detection of *Giardia* is also possible with *Giardia*-specific coproantigen detection assays [35, 48]. However, when not used in conjunction with a traditional microscopic technique, antigen testing may provide a false positive result in an animal that is no longer infected with *Giardia* due to persistent antigen excretion for several weeks or even months after parasite elimination [52, 53]. Given the shortcomings of current in-house diagnostic methods for *Giardia*, utilizing a deep learning algorithm platform, such as the VETSCAN IMAGYST system, could provide clinicians with an excellent additional or alternative diagnostic tool to help identify *Giardia* cases that would otherwise be missed.

Evaluation of the performance of the VETSCAN IMAGYST centrifugal flotation sample preparation method was limited in this study due to the modest numbers of true positives for the four targeted parasites and the inherent subsampling variability in non-homogenous fecal samples, which has been well documented in previous publications [54]. Kochanowski et al. [54] observed a wide range of coefficients of variation, between 31 and 254%, for *Toxocara* and *Trichuris* samples with a low number of egg counts, i.e. ≤ 50 EPG. Despite these limitations, the performance of the VETSCAN IMAGYST centrifugal flotation method in the present study was comparable to a conventional centrifugal flotation method, with diagnostic sensitivity and specificity of the comparisons ranging from 65.7 to 100% and 97.6–100%, respectively, across the four targeted parasites (Table 3). Additionally, one potential modification considered for the VETSCAN IMAGYST centrifugal flotation method to increase its diagnostic sensitivity is to lengthen the duration of centrifugation. Previous studies reported that egg recoveries with centrifugation at 264* × g* were significantly improved when the duration of centrifugation was increased from 1 and 3 min to 4 or 5 min at the same speed, although no change was observed in egg recovery when the time of centrifugation was extended to 10 or 20 min [37, 55].

As shown in Table 3, the diagnostic sensitivity and specificity of the VETSCAN IMAGYST centrifugal technique slightly surpassed those of the OVASSAY passive flotation method. Despite the fact that centrifugation significantly increases the sensitivity of fecal examinations, passive flotation continues to be the most commonly used technique in veterinary private practice due to its convenience [10, 19, 36, 51, 56–58]. Given that the VETSCAN IMAGYST system reliably recovers and detects parasite elements in fecal samples, does not largely depend on the experience level of examiners, and has previously been shown to provide results in around 10 min with the VETSCAN IMAGYST centrifugal flotation method [40], it has the potential to replace the conventional passive flotation method used in veterinary practice.

The most distinctive and unique feature of the VETSCAN IMAGYST system is its deep learning object detection algorithm. To the best of our knowledge, the VETSCAN IMAGYST system is the only automated diagnostic system that is integrated with a deep learning object detection algorithm and applied to veterinary medicine. Compared to shallow learning systems, which do not have any structural information on the function to be learned, deep learning algorithms exploit the advantage of locality at each level of the layered hierarchy, enabling the system to ignore the aspects that make computer vision brittle [59, 60]. The layered hierarchy also facilitates the system to continuously adapt to new data and apply these to new output classes with fewer examples [60, 61]. The deep learning characteristic, along with the YOLOv3 object detection model [43], which incorporates localization and classification features, results in a decrease of background errors and high agreement between the VETSCAN IMAGYST system and expert examinations. Another benefit of this system is its ability to store images and reports on a secure, cloud-based server system, allowing easy sharing of information by parasitologists as well as members of the veterinary and academic communities for patient care, research, and teaching.

The present study did not evaluate the usability of the VETSCAN IMAGYST system; however, our previous analysis showed that the VETSCAN IMAGYST system with the VETSCAN IMAGYST centrifugal flotation method could produce results in around 10 min, which is comparable to conventional fecal flotation tests. This time frame included the time to prepare the sample, i.e. approximately 3.5 min including the 2-min centrifugal incubation time, and the time to scan the images, i.e. approximately 6–7 min [40]. Data from the present study add to the body of evidence demonstrating the performance of the VETSCAN IMAGYST system in detecting intestinal parasite elements recovered from fecal samples. In addition to identifying the protozoan parasites *Cystoisospora* and *Giardia*, results from our present and previous studies show the system’s reliable performance in detecting four different genera/group of gastrointestinal parasites (*Ancylostoma*, *Toxocara*, *Trichuris*, Taeniidae) in dogs and cats [40]. With further training, the VETSCAN IMAGYST system will gain the ability to identify other parasites. The quantitative capability of the VESTSCAN IMAGYST system is currently under development. It is predicted that the algorithm will be able to perform a fecal egg counting test in the future.

## Conclusions

The VETSCAN IMAGYST system effectively recovered and identified feline *Ancylostoma* eggs, *Toxocara cati* eggs, *Cystoisospora* oocysts, and *Giardia* cysts in feline and canine fecal samples. Given the deep learning nature of the VETSCAN IMAGYST system, its performance is expected to improve over time, enabling it to be utilized in veterinary clinics to perform fecal examinations both accurately and efficiently.

## Data Availability

All the data generated or analyzed during this study are included in this published article.
